# Structure-Activity Relationship Studies of N- and C-Terminally Modified Secretin Analogs for the Human Secretin Receptor

**DOI:** 10.1371/journal.pone.0149359

**Published:** 2016-03-01

**Authors:** Kailash Singh, Vijayalakshmi Senthil, Aloysius Wilfred Raj Arokiaraj, Jérôme Leprince, Benjamin Lefranc, David Vaudry, Ahmed A. Allam, Jamaan Ajarem, Billy K. C. Chow

**Affiliations:** 1 School of Biological Sciences, The University of Hong Kong, Pokfulam Road, Hong Kong SAR, China; 2 Laboratory of Neuronal and Neuroendocrine Differentiation and Communication, Neurotrophic Factors and Neuronal Differentiation Team, Inserm U982, Associated International Laboratory Samuel de Champlain, Regional Platform for Cell Imaging of Haute-Normandie (PRIMACEN), University of Rouen, Mont-Saint-Aignan, France; 3 Department of Zoology, College of Science, King Saud University, Riyadh 11451, Saudi Arabia; 4 Department of Zoology, Faculty of Science, Beni-Suef University, Beni-Suef, Egypt; University of Oslo, NORWAY

## Abstract

The pleiotropic role of human secretin (hSCT) validates its potential use as a therapeutic agent. Nevertheless, the structure of secretin in complex with its receptor is necessary to develop a suitable therapeutic agent. Therefore, in an effort to design a three-dimensional virtual homology model and identify a peptide agonist and/or antagonist for the human secretin receptor (hSR), the significance of the primary sequence of secretin peptides in allosteric binding and activation was elucidated using virtual docking, FRET competitive binding and assessment of the cAMP response. Secretin analogs containing various N- or C-terminal modifications were prepared based on previous findings of the role of these domains in receptor binding and activation. These analogs exhibited very low or no binding affinity in a virtual model, and were found to neither exhibit *in vitro* binding nor agonistic or antagonistic properties. A parallel analysis of the analogs in the virtual model and *in vitro* studies revealed instability of these peptide analogs to bind and activate the receptor.

## Introduction

GPCRs are one of the largest receptor families [1]; these receptors share features in their molecular structure and signaling mechanisms and are regulated by a wide range of ligands such as hormones, peptides, neurotransmitters, chemokines, etc. GPCRs serve as the most important link between extracellular conditions and intracellular responses and are involved in most aspects of physiological processes [2]. Among GPCRs, the class B secretin receptor family has been found to mediate a broad array of homeostatic functions hence, represents putative drug target. Class B ligands such as calcitonin, glucagon and parathyroid hormone are currently used as therapeutic agents [3]. Secretin is a 27-residue linear peptide that is widely expressed throughout the body [4]. Secretin receptors are present in central and peripheral tissues [5], with an established role in the gastro-intestinal tract in which it regulates intraduodenal pH [6], acid release, stomach motility [7] and insulin secretion [8]. Recently, the roles of secretin in water balance [9], motor function [10], lipid homeostasis [11, 12], and appetite regulation [13] have also been demonstrated. This integrated role in physiology makes secretin a potential target for the treatment of metabolic disorders. The lack of structural insights into the interaction of hSR and secretin peptide remains the primary obstacle in the development of secretin agonist/antagonist and using the natural ligand as a therapeutic agent has not been feasible because of the short half-life of the peptide. Previously, most studies have investigated the physiological role of secretin, without much knowledge about the structure of its receptor [14]. The structures of some class A [15, 16] (http://tools.gpcr.org/crystalstructure/table) and class C [17, 18] GPCRs have been determined, and the entire structures of class B receptors [19, 20] have only been recently elucidated. These structural studies have led to an understanding of the active and inactive receptor conformations. Instead of distinct pharmacophores which are generally seen in ligands, the class B GPCR receptor ligands have a distributed interaction interface with its receptor [21]. It is also established that the extracellular C-terminal (Ct) region of the ligand is necessary for initial binding to the receptor and is also responsible for the specificity and allosteric activity of the receptor. In contrast, the extracellular N-terminal (Nt) region of the ligand is involved in secondary binding with the extracellular loop region and is responsible for downstream signaling [22–24]. Based on this information new molecules were designed in this study with modifications in the Nt region, whereas secretin from various non-mammalian vertebrates that contain variations in the Ct region were used as Ct-modified analogs to assess their effect on the human secretin receptor (hSR). In the absence of an experimentally determined structure for hSR, a homology-modeled 3D receptor structure was developed to provide additional details on the receptor-ligand interaction. These secretin analogs were studied in parallel with virtual docking, *in vitro* binding and functional assays to investigate their interaction with the hSR.

## Experimental Methods

### Materials

Human secretin, human glucagon, and secretin analogs 1–5 and 15–20 of greater than 95% purity were purchased from GenScript, USA. The SNAP-tag vector (PLASCUST), Tag-lite^®^ labeling media (SSNPTBX), and Lumi4-Tb (SSNPTBD) were purchased from Cisbio, USA. MEM media (61100–061), Versene (15040–066) and HBSS buffer (14025134) were purchased from Gibco^®^, Life Technologies. Primers were custom designed and purchased from Invitrogen. The HTRF-LANCE^®^ cAMP assay kit (AD0262) was obtained from PerkinElmer. The 384-well black plates were purchased from (Greiner Bio-One, 788086). The MOE software was licensed through Cloud Scientifics, China. Schrodinger software was licensed from Schrödinger LLC.

### Secretin peptide analogs

Different secretin peptide analogs were designed by using the primary amino acid of human secretin mature peptide:

H_2_N-His-Ser-Asp-Gly-Thr-Phe-Thr-Ser-Glu-Leu-Ser-Arg-Leu-Arg-Glu-Gly-Ala-Arg-Leu-Gln-Arg-Leu-Leu-Gln-Gly-Leu-Val-COOH.

The following secretin peptide analogs were evaluated in this study:

1. h[Pro^2^]SCT:
  H_2_N-His-Pro-Asp-Gly-Thr-Phe-Thr-Ser-Glu-Leu-Ser-Arg-Leu-Arg-Glu-Gly-Ala-Arg-Leu-Gln-Arg-Leu-Leu-Gln-Gly-Leu-Val- COOH
2. h[Ala^1^]SCT:
  H_2_N-Ala-Ser-Asp-Gly-Thr-Phe-Thr-Ser-Glu-Leu-Ser-Arg-Leu-Arg-Glu-Gly-Ala-Arg-Leu-Gln-Arg-Leu-Leu-Gln-Gly-Leu-Val- COOH
3. h[Leu^1^]SCT:
  H_2_N-Leu-Ser-Asp-Gly-Thr-Phe-Thr-Ser-Glu-Leu-Ser-Arg-Leu-Arg-Glu-Gly-Ala-Arg-Leu-Gln-Arg-Leu-Leu-Gln-Gly-Leu-Val- COOH
4. h[(*p*-Cl,D-Phe^4^]SCT:
  H_2_N-His-Ser-Asp-p-Cl,D-Phe^4^-Thr-Phe-Thr-Ser-Glu-Leu-Ser-Arg-Leu-Arg-Glu-Gly-Ala-Arg-Leu-Gln-Arg-Leu-Leu-Gln-Gly-Leu-Val- COOH
5. h[(D-allyl,Gly^4^]SCT:
  H_2_N-His-Ser-Asp-D-allyl,Gly-Thr-Phe-Thr-Ser-Glu-Leu-Ser-Arg-Leu-Arg-Glu-Gly-Ala-Arg-Leu-Gln-Arg-Leu-Leu-Gln-Gly-Leu-Val- COOH
6. hSCT_(6–27)_:
  H_2_N-Phe-Thr-Ser-Glu-Leu-Ser-Arg-Leu-Arg-Glu-Gly-Ala-Arg-Leu-Gln-Arg-Leu-Leu-Gln-Gly-Leu-Val- COOH
7. h[Cha^4^]SCT:
  H_2_N-His-Ser-Asp-Cha-Thr-Phe-Thr-Ser-Glu-Leu-Ser-Arg-Leu-Arg-Glu-Gly-Ala-Arg-Leu-Gln-Arg-Leu-Leu-Gln-Gly-Leu-Val- COOH
8. h[D-Asp^3^]SCT:
  H_2_N-His-Ser-D-Asp-Gly-Thr-Phe-Thr-Ser-Glu-Leu-Ser-Arg-Leu-Arg-Glu-Gly-Ala-Arg-Leu-Gln-Arg-Leu-Leu-Gln-Gly-Leu-Val- COOH
9. h[Tic^1^]SCT:
  H_2_N-Tic-Ser-Asp-Gly-Thr-Phe-Thr-Ser-Glu-Leu-Ser-Arg-Leu-Arg-Glu-Gly-Ala-Arg-Leu-Gln-Arg-Leu-Leu-Gln-Gly-Leu-Val- COOH
10. h[Cit^1^]SCT:
  H_2_N-Cit-Ser-Asp-Gly-Thr-Phe-Thr-Ser-Glu-Leu-Ser-Arg-Leu-Arg-Glu-Gly-Ala-Arg-Leu-Gln-Arg-Leu-Leu-Gln-Gly-Leu-Val- COOH
11. h[*p*CO_2_H-Phe^3^]SCT:
  H_2_N-His-Ser-pCO2HPhe-Gly-Thr-Phe-Thr-Ser-Glu-Leu-Ser-Arg-Leu-Arg-Glu-Gly-Ala-Arg-Leu-Gln-Arg-Leu-Leu-Gln-Gly-Leu-Val- COOH
12. h[Orn^1^]SCT:
  H_2_N-Orn-Ser-Asp-Gly-Thr-Phe-Thr-Ser-Glu-Leu-Ser-Arg-Leu-Arg-Glu-Gly-Ala-Arg-Leu-Gln-Arg-Leu-Leu-Gln-Gly-Leu-Val- COOH
13. h[Pro^4^]SCT:
  H_2_N-His-Ser-Asp-Pro-Thr-Phe-Thr-Ser-Glu-Leu-Ser-Arg-Leu-Arg-Glu-Gly-Ala-Arg-Leu-Gln-Arg-Leu-Leu-Gln-Gly-Leu-Val- COOH
14. h[Tyr^4^]SCT:
  H_2_N-His-Ser-Asp-Tyr-Thr-Phe-Thr-Ser-Glu-Leu-Ser-Arg-Leu-Arg-Glu-Gly-Ala-Arg-Leu-Gln-Arg-Leu-Leu-Gln-Gly-Leu-Val- COOH
15. Rat SCT
  H_2_N-His-Ser-Asp-Gly-Thr-Phe-Thr-Ser-Glu-Leu-Ser-Arg-Leu-Gln-Asp-Ser-Ala-Arg-Leu-Gln-Arg-Leu-Leu-Gln-Gly-Leu-Val- COOH
16. Xenopus SCT
  H_2_N-His-Val-Asp-Gly-Arg-Phe-Thr-Ser-Glu-Phe-Ser-Arg-Ala-Arg-Gly-Ser-Ala-Ala-Ile-Arg-Lys-Ile-Ile-Asn-Ser-Ala-Leu-Ala- COOH
17. Chicken SCT
  H_2_N-His-Ser-Asp-Gly-Leu-Phe-Thr-Ser-Glu-Tyr-Ser-Lys-Met-Arg-Gly-Asn-Ala-Gln-Val-Gln-Lys-Phe-Ile-Gln-Asn-Leu-Met- COOH
18. Coelacanths SCT
  H_2_N-His-Val-Asp-Gly-Leu-Phe-Thr-Ser-Glu-Leu-Ser-Lys-Leu-Arg-Gly-Ser-Ala-Val-Ala-Arg-Ser-Phe-Thr-Asn-Ala-Val-Leu- COOH
19. Nt-SCT
  H_2_N-His-Ser-Asp-Gly-Thr-Phe-Thr-Ser-Glu-Leu-Ser-Arg-Leu- COOH
20. Ct-SCT
  H_2_N-Arg-Glu-Gly-Ala-Arg-Leu-Gln-Arg-Leu-Leu-Gln-Gly-Leu-Val- COOH
21. (19 + 20) (combinatorial treatment–both Nt and Ct analyzed as a cotreatment)

### Chemicals and reagents

All Fmoc-amino-acid residues, *O*-benzotriazol-1-yl-*N*,*N*,*N*,*N*'-tetramethyluronium tetrafluoroborate (TBTU), and 1-hydroxybenzotriazole (HOBt) were purchased from PolyPeptide Laboratories (Strasbourg, France), Novabiochem Merck Chemicals (Nottingham, UK) or Christof Senn Laboratories (Dielsdorf, Switzerland). Preloaded 4-hydroxymethyl-phenoxymethyl-copolystyrene-1%-divinylbenzene resin (Fmoc-Val-HMP) was obtained from Life Technologies (Villebon sur Yvette, France). *N*,*N*-Diisopropylethylamine (DIEA), piperidine, trifluoroacetic acid (TFA), and triisopropylsilane (TIS) were obtained from Acros Organics (Geel, Belgium). *N*-Methylpyrrolidone (NMP), dichloromethane (DCM) and other reagents were purchased from Sigma-Aldrich (Saint-Quentin-Fallavier, France). Alexa Fluor^®^ 488 C_5_ maleimide thiol-selective dye was obtained from Life Technologies. Acetonitrile was purchased from Fisher Scientific (Illkirch, France). hSCT_(6–27)_, h[Tic^1^]SCT, h[Cit^1^]SCT, h[Orn^1^]SCT, h[D-Asp^3^]SCT, h[*p*CO_2_H-Phe^3^]SCT, h[Pro^4^]SCT, h[Tyr^4^]SCT and h[Gly^28^, Cys^29^]SCT were synthesized as previously described [25]. Briefly, the hSCT analogs were synthesized (0.1-mmol scale) by solid phase methodology on an Fmoc-Val-HMP or an Fmoc-Cys(Trt)-HMP resin using a 433A Applied Biosystems peptide synthesizer (Applera-France, Courtaboeuf, France) and the standard Fmoc manufacturer’s procedure. All Fmoc-amino-acids (1 mmol, 10 eq.) were coupled by *in situ* activation with TBTU/HOBt (1.25 mmol: 1.25 mmol, 12.5 eq.) and DIEA (2.5 mmol, 25 eq.) in NMP. Peptides were deprotected and cleaved from the resin by adding 10 mL of TFA/TIS/H_2_O (99.5:0.25:0.25, v/v/v) for 120 min at room temperature. After filtration, crude peptides were precipitated by the addition of tert-butyl methyl ether (TBME), centrifuged (4,500 rpm), washed twice with TBME, and lyophilized. The synthetic peptides were purified by reversed-phase HPLC on a 2.2 x 25 cm Vydac 218TP1022 C_18_ column (Grace, Epernon, France) using a linear gradient (10–50% over 45 min) of acetonitrile/TFA (99.9:0.1; v/v) at a flow rate of 10 mL/min. Analytical HPLC was performed using a 0.46 x 25 cm Vydac 218TP54 C_18_ column (Grace) and indicated that the purity of all peptides was >99.1%. The purified peptides were characterized by MALDI-TOF mass spectrometry on a Voyager DE PRO (Applera-France) in the reflector mode using ɑ-cyano-4-hydroxycinnamic acid as the matrix and peptides of known molecular mass for calibration. h[Gly^28^, Cys^29^]SCT (6.2 mg, 1.4 eq.) was dissolved in 10 mM phosphate buffer (8 mL, pH 7.4) at room temperature. Alexa Fluor^®^ 488 C_5_ maleimide thiol-selective dye (1 mg, 1 eq.) was dissolved in 1 mL of water and added dropwise to the h[Gly^28^, Cys^29^]SCT solution. The reaction was monitored by RP-HPLC until completion, and the reaction medium was lyophilized. The Cys^29^-conjugate h[Gly^28^, Alexa-Cys^29^]SCT was purified and characterized as previously described [25].

### Homology modeling

The 3D model of hSR was prepared by modeling the Nt and the trans membrane (TM) region separately. The Nt model was prepared using multiple templates like Nt region of the PACAP receptor (PAC1) (PDB ID: 2JOD) [26] (Table 1), Nt region of the VPAC-2 receptor (PDB ID: 2X57) at 2.10 Å resolution [27], the Nt region of the GLP-1 receptor (PDB ID: 3C5T) at 2.10 Å [28] *etc*. All the initial models were then evaluated by SAVES server (http://services.mbi.ucla.edu/SAVES/) [29] and the model based on the template of Nt extracellular domain of human pituitary adenylate cyclase 1 receptor (PDB ID: 3N94) **(Figure A in** S1 File**)** was found to be the best structure [30] with sequence identity of 46.43 percent **(Figure A in** S1 File**)**. The TM backbone model was generated using the crystal structures of the glucagon receptor (PDB ID: 4L6R) at 3.30 Å resolution [20] and corticotropin-releasing factor receptor 1 (PDB ID: 4K5Y) at 2.98 Å resolution [19] **(Figure B in** S1 File**)**. The structure of the glucagon receptor (GCGR) with sequence similarity of 49.63 percent was used as the primary template for the TM region. The alignments were performed using the details available from Uniprot C [31] by fixing the residues and regions according to sequence and structural similarity. The developed model was validated for structural orientation and disulfide bonds. After validation, the Nt and TM were fused at the over-hanged region **(Figure C in** S1 File**)** with the help of rigid docking by Pydock [32] to produce 102 models which were later screened for correct orientation of EC domain and TM region by structural validation, the tertiary structure of the receptor was compared with other class B templates [24] reducing the suitable models to 28 and on further screening 5 models were observed to have the best ERRAT score (crystallographic errors) [33]. The best model was selected based on the docking score between the receptor and ligands (hSCTF, human vasoactive peptide (hVIP), human gastric inhibitory polypeptide (hGIP) and human pituitary adenylate cyclase-activating polypeptide (hPACAP). The TM region was validated by the help of ProQM which is the only model quality assessment algorithm for membrane protein [34]. The 3-D models of hGIP, hVIP and hPACAP were retrieved from the RCSB protein data bank 2OBU [35], 2RRH [36] and 2D2P (yet to be published), respectively. The homology model of secretin and its analogs were also generated from solution NMR structures using hPACAP (PDB ID: 2D2P) and, crystal structure of glucagon (PDB ID: 3IOL) [37]. The 3D structures of analog number 5, 7, 9, 10 and 12 were not modeled due to unavailability of a template with non-standard amino acids.

**Table 1:** Table showing percentage sequence similarity by pairwise alignment of the amino acid sequences with Nt and TM region of hSR.

Protein name	PDB ID	%sequence similarity of N-terminal region.	%sequence similarity of TM region
Pituitary adenylate cyclase-activating polypeptide type I receptor	2JOD	44.44	-
Vasoactive intestinal polypeptide receptor 2	2X57	33.00	-
Glucagon-like peptide 1 receptor	3C5T	27.19	-
Pituitary adenylate cyclase 1 Receptor	3N94	46.43	-
Soluble cytochrome b562 and Glucagon receptor chimera	4L6R	-	51.91
Corticotropin-releasing factor receptor 1, T4-Lysozyme chimeric construct	4K5Y	-	35.63

### Virtual docking

The docking stimulation was performed using Schrödinger biological suite [38] and patchdock/firedock was used to estimate the docking score [39]. The refined and validated receptor and ligand models were used for virtual docking. The receptor was analyzed for possible binding sites using Schrödinger biological suite for docking between hSCT and hSR models. Using hSCT, hVIP as positive control and GIP and others as negative control, ligand model was first docked with the five receptor models with the best ERRAT score in order to find the best docking site between hSCT and hSR EC domain [40]. The best model was used to calculate the docking score with all the secretin analogs by patchdock/firedock. The peptide and the protein were docked by patchdock and refinement was performed by firedock, the top 10 models after firedock refinement were analyzed. The binding energy of the best-docked model (among the top ten) at the correct binding site was used for affinity analysis. If the top ten structure with the ligand at correct binding site were not available, structure with the highest delta G score was used.

### FRET binding assay

A non-radioactive binding assay was used to evaluate the binding affinity of the analogs [41]. SNAPtag-hSR-transfected CHO-K1 cells expressed the hSR at the plasma membrane with a SNAP-tag at the Nt region of the receptor. The cells were washed, and the SNAPtag-hSR was labeled with 100 nM Lumi4Tb at 37°C for 1 h. The h[Gly^28^, Alexa-Cys^29^]SCT analog was used as an acceptor. The labeled cells were detached with Versene and counted. In 384-well black plates, 10,000 cells in 5 μL of labeling media, 5 μL of labeling media, 5 μL of labeled hSCT (final concentration of 500 nM), and 5 μL of the peptide analogs (final concentration of 10 μM) were added to each well. The microplate was centrifuged and incubated at 4–8°C for 1 h. Unlabeled hSCT and hGLU were used as positive and negative controls, respectively. Peptide analogs that exhibited binding at a concentration of 10 μM were evaluated for a dose response ranging from 10^−12^ M to 10^−4^ M to determine the IC_50_ value. The nonspecific FRET signals were measured with 100 μM unlabeled hSCT at respective concentrations of peptide analogs and was corrected from the total binding. The absorbance was measured at 615 and 520 nm in a VICTOR^™^ X4 spectrofluorometer (PerkinElmer), and the FRET signals were analyzed.

### Functional assays

#### Agonist-cAMP response

The cAMP responses in hSR-transfected CHO-K1 cells were detected using the HTRF-LANCE^®^ cAMP assay kit. After 48 h of transfection, the cells were detached by nonenzymatic treatment using Versene. The cells were counted and processed according to the manufacturer’s instructions to measure the cAMP response following cell treatment with the secretin analogs. hSCT was used as a positive control. The TRF signals (340 nm excitation/665 nm emission) were detected using a VICTOR X4 spectrofluorometer (PerkinElmer), and the cAMP concentration was determined using a standard curve.

#### Antagonist-cAMP response

The antagonistic properties of the peptide analogs were analyzed by coincubation of hSCT with increasing concentrations of the analogs, and the changes in the cAMP responses were measured using the HTRF-LANCE^®^ cAMP assay kit. The detached cells were diluted in the stimulation buffer with Alexa Fluor^®^ 647-conjugated anti-cAMP antibody, and 10,000 cells per well (10 μL) were seeded in 384-well microplates and incubated with 5 μL of 5 nM hSCT plus 5 μL of the peptide analogs (10^−12^ to 10^−6^ M) for 30 min. hSCT was used as a positive control for agonist activity. Detection was subsequently performed according to the manufacturer’s instructions.

### Statistical analysis

All *in vitro* results were analyzed in quadruplicate, and the data are expressed as the means ± SEM. Significant differences were identified using Student’s t-test at p<0.05. The dose response was analyzed using GraphPad Prism 5.0 software with a variable slope (four parameters).

## Results

### Model building

**Fig 1:**
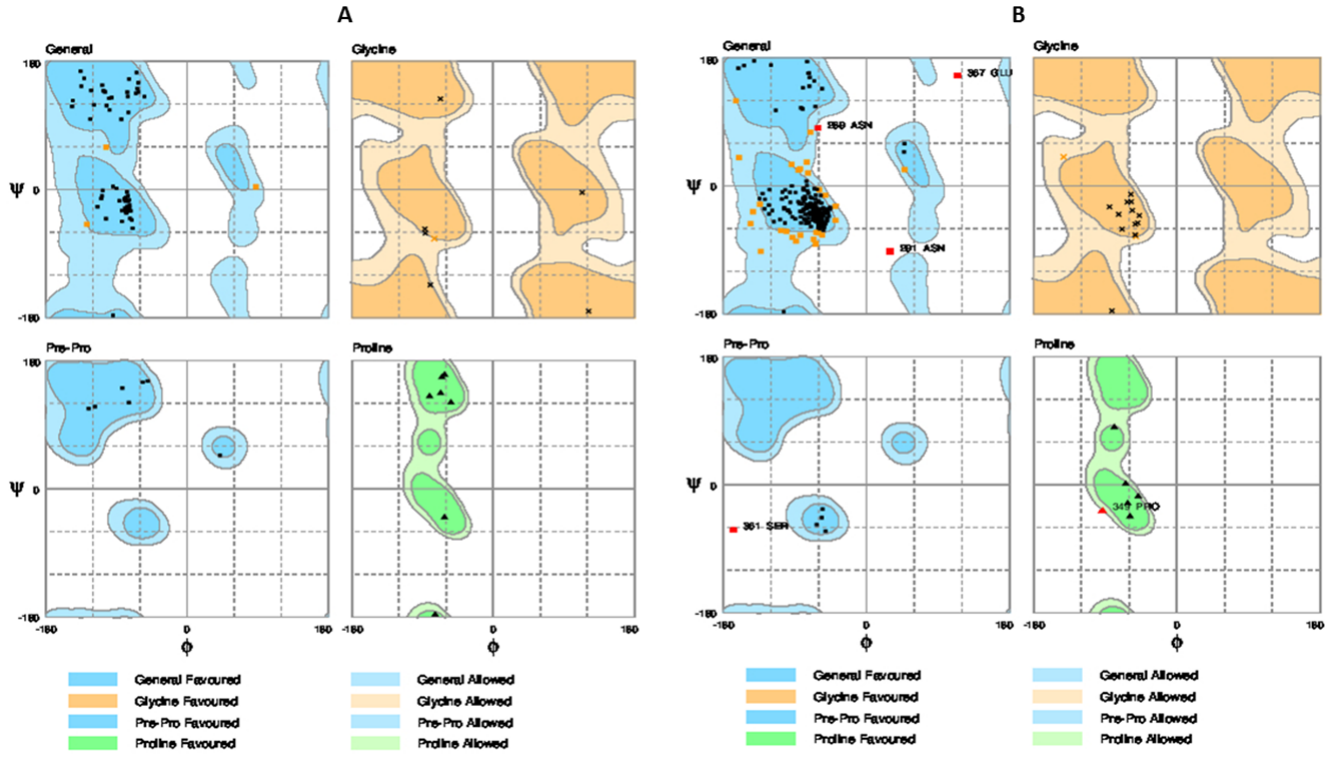
Ramachandran plot structural validation by RAMPAGE of A) Nt sequence with zero residue in disallowed region and B) TM region shows only five outlier residues. The detailed mapping of outliers are explained in Table 2.

**Fig 2:**
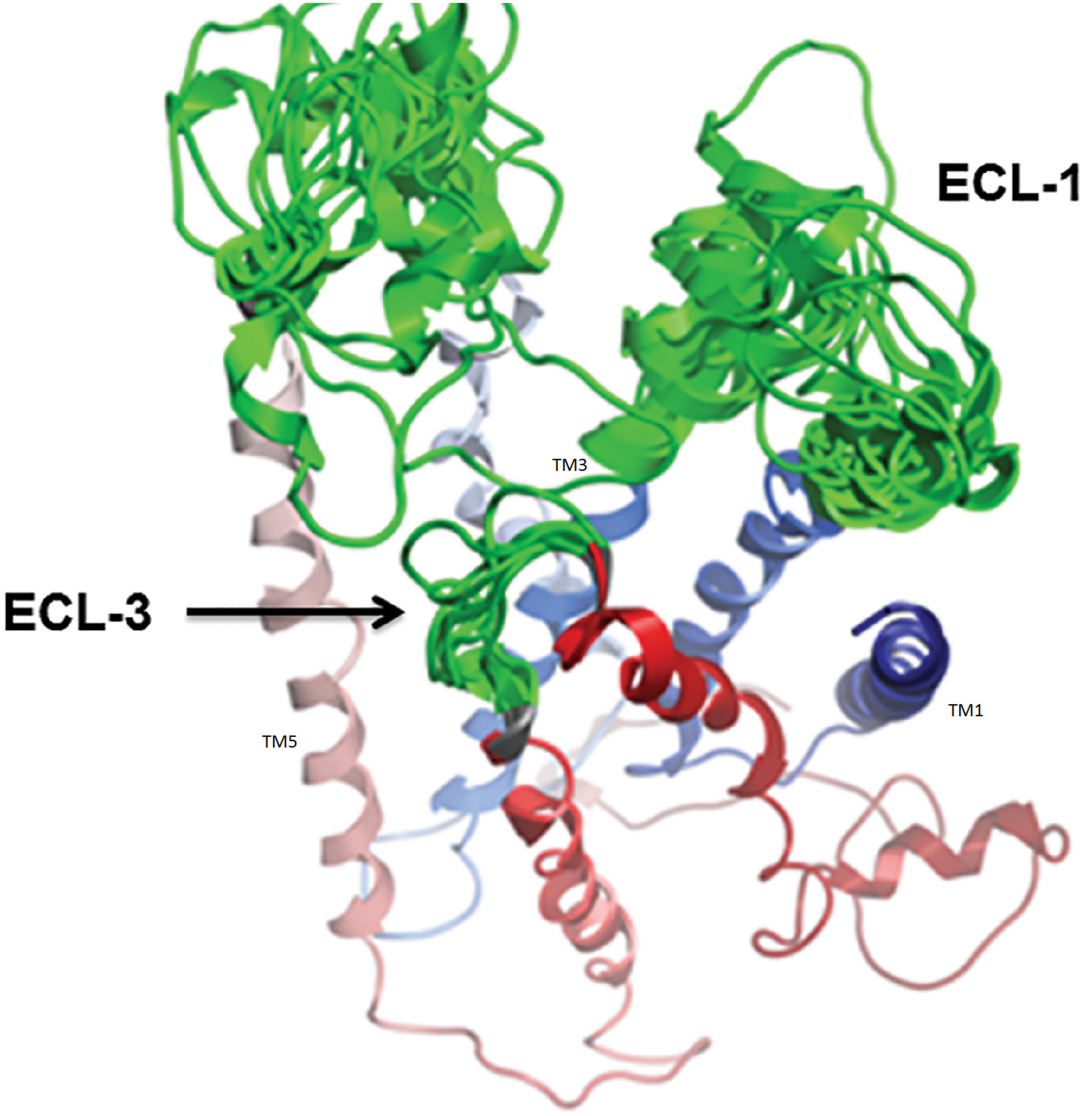
Comperitive loop modeling of the ECL region. The best fit loop with reduced outliers is chosen.

**Fig 3:**
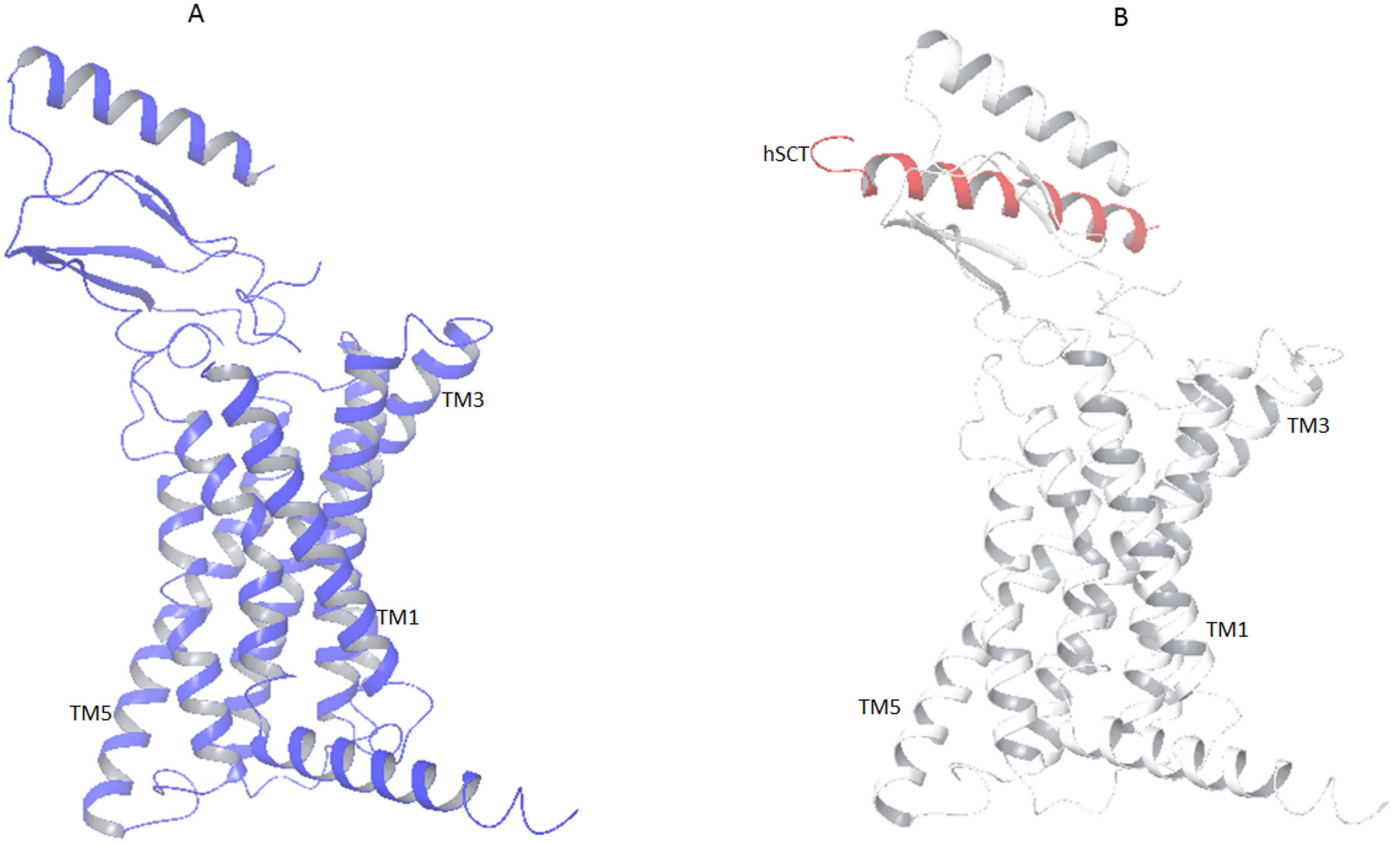
A)The final 3D model generated and refined using multiple templates. B) The model with docked hSCT (red) shows the binding site at the Nt of the receptor.

**Fig 4:**
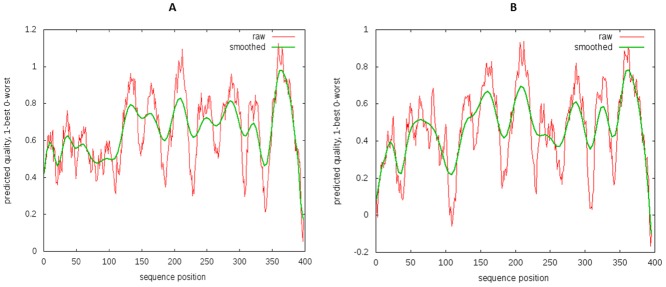
The Model quality assessment for membrane protein A) Positive control 4L6R B) The ProQM of full receptor model showing preferred quality value to be higher than 0.4 for TM region (after 120 amino acid residue).

The complete 3 dimensional model of hSR was created by *in silico* homology modeling approach. As there is no direct template available for 3D modeling of full hSR, the Nt domain was modeled with a multiple template approach separately from TM. First the Nt domain was modeled with multiple templates and the best model was selected with the help of SAVES (server analysis for model validation). The Ramachandran plot of the final model shows zero residues in the outlier region by rampage [42] (Fig 1A). Verify 3D [43] results showed 81.18% to have 3D-1D score > = 0.2. The TM region was then modeled using GCGR crystal structure as the template. The template query alignment was performed without gaps in the helices and gaps reduced to one in the loop region. These gaps were separately modeled using comparative loop modeling **(**Fig 2**)**. The structural geometry was validated with Ramachandran plot and through confirmation of the beta sheet and helical conformation for the predicted backbone [42, 44–46] **(**Fig 1B**)**. The position of each outlier was analyzed and was found to be negligible (Table 2). The model of the Nt and TM were fused by rigid body docking using Pydock [47] **(**Fig 3**)**. The best five models from a total of 102 were screened for structural validity. The tertiary structure of the receptor was screened by comparison with other class B templates [24] and by considering the best ERRAT value. This models were screened by ProQM which is the only model quality assessment algorithm for membrane protein **(**Fig 4**)**. The binding site of SCT on to the receptor was determined by Schrodinger protein-protein docking algorithm **(**Fig 3B). The docking site was confirmed using binding energy estimation by docking the receptor with hVIP, hPACAP and hGIP (Table 3).

**Table 2:** Ramachandran plot outliers.

S. No	Residue (Outlier)	Region
Fle1	269 Asn	TM 4 helical region
2	367 Glu	ECL6
3	291 Asn	ECL4
4	361 Ser	TM 6 helical region
5	367 Glu	ECL3

Less than 2% were outliers; all the outliers were in insignificant positions when docking.

**Table 3:** Virtual docking: Validation.

Peptide	Total binding energy	Binding affinity	*In vitro* IC_50_
hSCT	-11.53	++++	1.630 + 3.55 nM
hVIP	-9.51	+++	3.082 + 1.06 μM
hPACAP	-6.31	++	-
hGIP	+10.63	-	-

Both hSCT and hVIP exhibit binding affinity in the virtual model and in the in vitro assay. In the in vitro assay, hPACAP binds at only high concentrations, which indicates weak binding affinity in the virtual docking.

### Model verification by *in vitro* assay

**Fig 5:**
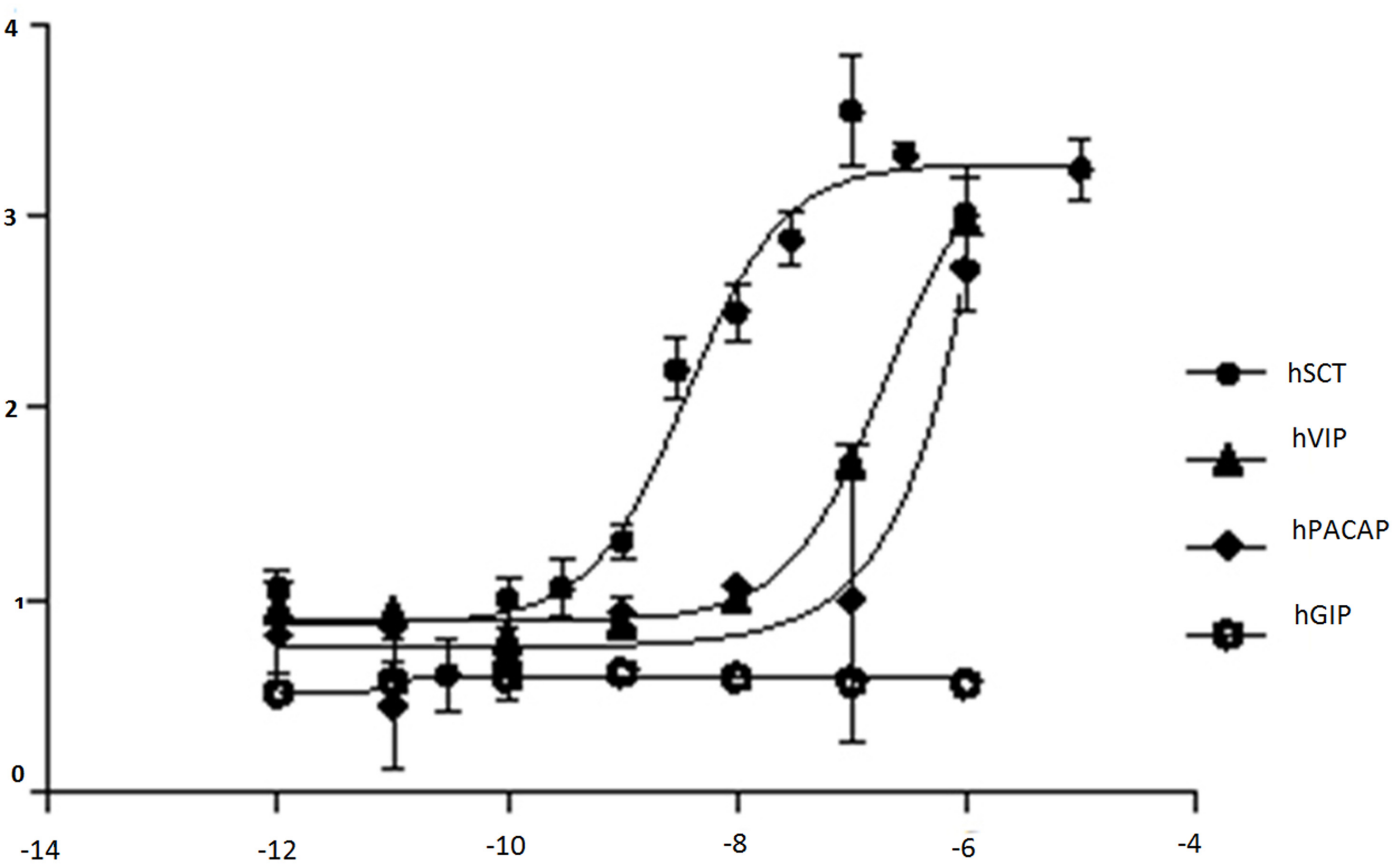
cAMP assay for different peptides ligands to check the activation of the receptor were hSCT acts as a positive control.

**Fig 6:**
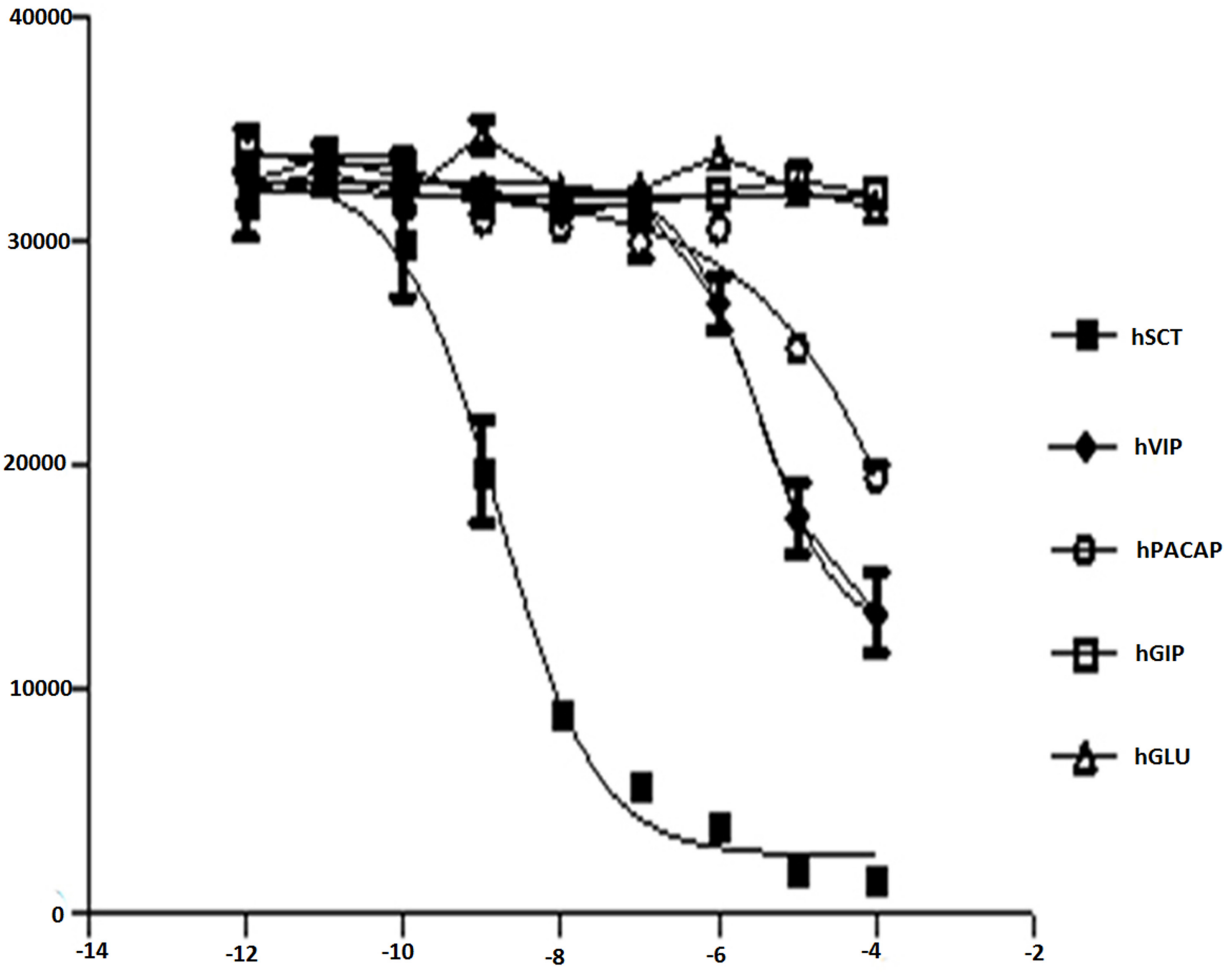
FRET binding–dose response of the active analog 15. hSCT served as the positive control, and hGLU served as the negative control. Rat SCT indicates analog 15.

The model was validated by verifying the docking score and by *in vitro* cAMP (Fig 5) and FRET binding assays (Fig 6). The assay results were consistent with the docking results where hSCT was used as a positive control. Peptides like hVIP, hPACAP were found to exhibit some binding affinities in docking analyses could also activate hSR to produce cAMP, and their interactions with hSR were also confirmed by the FRET assay. The binding energy of hSCT, hVIP and hPACAP were -11.53, -9.51 and -6.31, respectively, while hGIP has a binding energy of +10.63, clearly indicates its inability to interact with hSR.

### Virtual docking and cAMP studies on hSCT analogs

**Fig 7:**
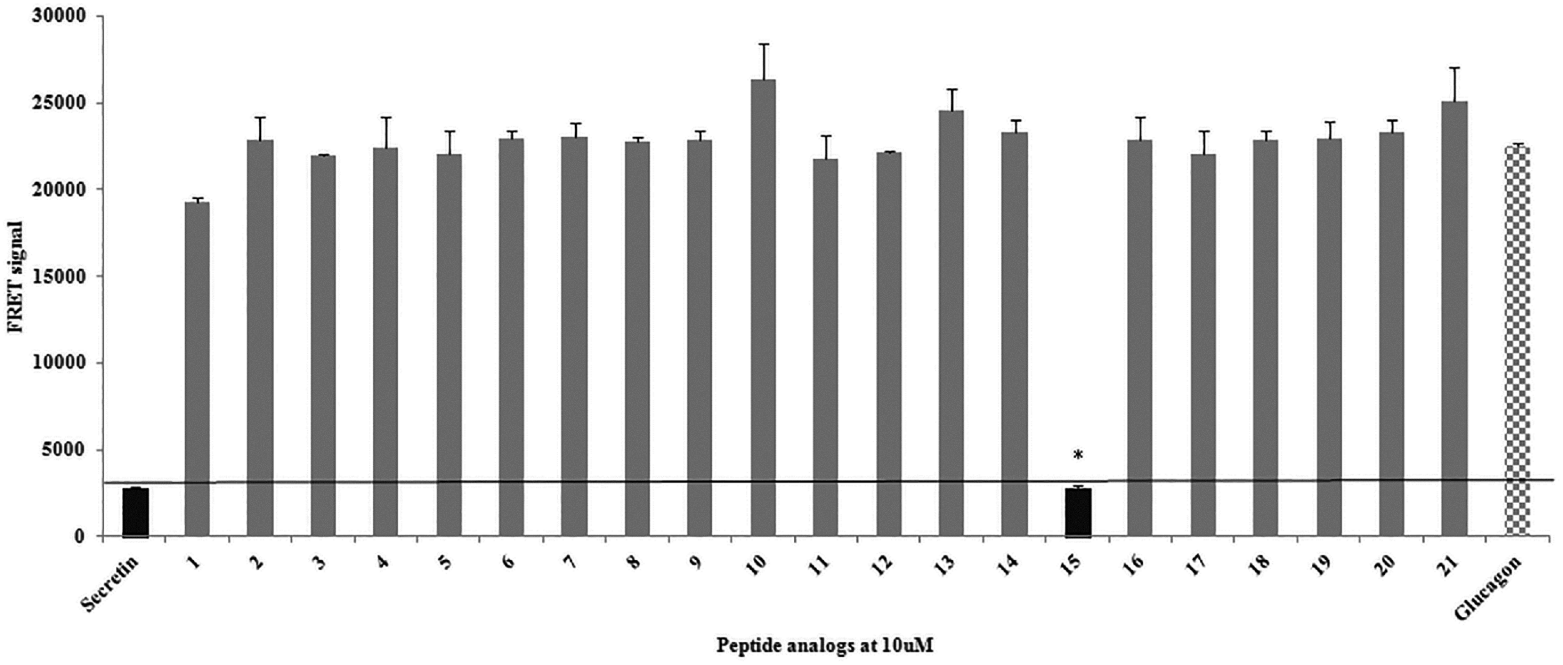
The binding efficiency of peptide analogs at 10 μM in the FRET competitive binding assay. High FRET signals indicate no binding, whereas low FRET signals indicate binding. Peptide analog 15 exhibits significant binding affinity at 10 μM, in contrast to the remaining analogs. *, p < 0.0005.

Structures of hSCT analogs were prepared by homology modeling and were used for virtual docking with hSR. Functional cAMP assays were performed in parallel to confirm the docking result and to test the ability of any of these analogs acting as agonist or antagonist. The best model was used to calculate the binding energy of these secretin analogs. All the analogs, with the exception of analog 15 (rat secretin) that showed very high delta G, exhibited low binding potential at the binding site (Table 4). The binding affinity of these SCT analogs were further evaluated, and only rat SCT was found to exhibit binding affinity for hSR (Fig 7) with an IC_50_ value of 0.40 ± 0.35 nM, which is higher than that of hSCT (1.6 ± 1.1 nM). The virtual docking and *in vitro* binding assay show positive correlation for class B ligands (Table 4). Through cAMP assays, only rat SCT was identified as an agonist of hSR, whereas all other SCT analogs exhibited neither agonistic nor antagonistic properties **(Figure D in** S1 File**)**.

**Table 4:** Comparison of virtual and *in vitro* results for the peptide analogs.

Analogs	Total binding energy	*In vitro* binding	Agonistic response	Antagonistic response
**hSCT**	**-11.53**	**Present**	**Present**	**Absent**
Analog 1	+7465.64	Unstable	Absent	Absent
Analog 2	> +1387.81	Unstable	Absent	Absent
Analog 3	> +1387.81	Unstable	Absent	Absent
Analog 4	> +12.18	Unstable	Absent	Absent
Analog 5	-	Unstable	Absent	Absent
Analog 6	+12.30	Unstable	Absent	Absent
Analog 7	-	Unstable	Absent	Absent
Analog 8	+4.69	Unstable	Absent	Absent
Analog 9	-	Unstable	Absent	Absent
Analog 10	-	Unstable	Absent	Absent
Analog 11	>1387.81	Unstable	Absent	Absent
Analog 12	-	Unstable	Absent	Absent
Analog 13	+1010.29	Unstable	Absent	Absent
Analog 14	>+10.24	Unstable	Absent	Absent
**Analog 15**	**-10.21**	**Present**	**Present**	**Absent**
Analog 16	-1.38	Absent	Absent	Absent
Analog 17	+14.45	Absent	Absent	Absent
Analog 18	+4.23	Absent	Absent	Absent
Analog 19	>+77.89	Unstable	Absent	Absent
Analog 20	>+3541.03	Unstable	Absent	Absent
Analog 21		Unstable	Absent	Absent

The analogs containing Nt and Ct modifications were screened for virtual interactions and exhibit affinity for the active receptor model, whereas in the in vitro assay with the receptor in the resting state, the analogs fail to bind or activate the receptor. Model of analog 5, 7, 9, 10 and 12 were not modeled due to unavailability of a template with non-standard amino acids.

## Discussion

The structural elucidation of membrane proteins, particularly GPCRs, is challenging. Therefore, homology modeling is a tempting alternative to generate a virtual 3D model of these receptors that can be used as a primary tool in virtual screening to understand the binding affinity against a library of analogs. GPCRs possess a similar topology and activation mechanism [48, 49]. In this study, we have used a multiple-template approach to generate a 3D model of the hSR. Both the Nt and the TM regions have been individually modeled using respective templates for the active state receptor conformations. The secondary structure of the final Nt-TM fused model was studied/analyzed for structural validity. The tertiary structure of the receptor was compared with other class B templates [24]. The structural geometry was assessed using Ramachandran plot (Fig 3), all of the five outliers were found to lie in the non-binding regions and the total outliers are below 2%. The model was further virtually confirmed by docking with hSCT and class B ligands and experimentally confirmed using biological assays. There has been an allosteric model proposed on receptor activation in class B GPCRs where the C terminal of the peptide hormone first interacts with the Nt domain of the receptor while the N-terminal of the ligand subsequently interacts with the extracellular TM loops [50–56]. It is also worth mentioning here that there is a different model for hSR activation. It was found that minor modification at the N-terminal of the secretin peptide resulted in no direct interaction of the ligand with the receptor TM loops, but binding of the ligand only with the Nt of the secretin receptor was sufficient to activate the receptor [57]. This observation suggests the presence of an agonist epitope hidden within the receptor Nt [58], and it was hypothesized that the binding of natural ligands to the conserved disulfide-bonded in the N-terminal domain of the receptor may lead to a conformational change in the N-terminal for receptor activation [59]. In virtual docking, hSCT, hPACAP and hVIP were found to bind hSR with high binding energy in respective order. *In vitro* experiments revealed that hPACAP and hVIP also bind to hSR and activate downstream signaling, but only at high concentrations. These results confirm the weak affinity of hPACAP and hVIP for hSR and support the specificity of our active receptor model, hence the model may serve as a tool to identify agonists or antagonists by targeting the active binding site. We have therefore used this hSR model for virtual docking of the peptide analogs that we have synthesized. We found those analogs with modifications in either the Nt or the Ct region exhibited very low or no binding affinity for the receptor. However, human and rat SCT exhibited strong ligand interactions with good docking scores. Frog, chicken and coelacanth SCT exhibited docking scores lower than the one observed for hSCT. To verify the *in silico* data, all these analogs were studied by *in vitro* FRET-based competitive binding assay. In this assay, FRET signals were detected only when the labeled Nt of the receptor and the Ct of the labeled ligand were in close proximity. In this study, only human and rat SCTs could bind to hSR (IC_50_ 1.6 ± 1.1 and 0.40 ± 0.35 nM, respectively). Frog, chicken, coelacanth SCT and all other SCT analogs did not exhibit binding affinity even at a high concentration (Fig 6). As GPCRs are known to possess more than one binding site, we have also tested the agonistic (Table 5) or antagonistic properties of these analogs by cAMP assays **(Figure D in** S1 File**)**, but none of these analogs exhibit activities excluding the possibility of allosteric binding for these modified peptide analogs, which is consistent with the virtual docking data.

**Table 5:** Pair wise sequence alignment of all analogs with hSCT.

hSCT|P09683	H	S	D	G	T	F	T	S	E	L	S	R	L	R	E	G	A	R	L	Q	R	L	L	Q	G	L	V
1analog	H	**P**	D	G	T	F	T	S	E	L	S	R	L	R	E	G	A	R	L	Q	R	L	L	Q	G	L	V
2analog	**A**	S	D	G	T	F	T	S	E	L	S	R	L	R	E	G	A	R	L	Q	R	L	L	Q	G	L	V
3analog	**L**	S	D	G	T	F	T	S	E	L	S	R	L	R	E	G	A	R	L	Q	R	L	L	Q	G	L	V
4analog	H	S	D	**F**	T	F	T	S	E	L	S	R	L	R	E	G	A	R	L	Q	R	L	L	Q	G	L	V
5analog	H	S	D	**G**	T	F	T	S	E	L	S	R	L	R	E	G	A	R	L	Q	R	L	L	Q	G	L	V
6analog						F	T	S	E	L	S	R	L	R	E	G	A	R	L	Q	R	L	L	Q	G	L	V
7analog	H	S	D	**C**	T	F	T	S	E	L	S	R	L	R	E	G	A	R	L	Q	R	L	L	Q	G	L	V
8analog	H	**P**	D	G	T	F	T	S	E	L	S	R	L	R	E	G	A	R	L	Q	R	L	L	Q	G	L	V
9analog	**T**	S	D	G	T	F	T	S	E	L	S	R	L	R	E	G	A	R	L	Q	R	L	L	Q	G	L	V
10analog	**C**	S	D	G	T	F	T	S	E	L	S	R	L	R	E	G	A	R	L	Q	R	L	L	Q	G	L	V
11analog	H	S	**F**	G	T	F	T	S	E	L	S	R	L	R	E	G	A	R	L	Q	R	L	L	Q	G	L	V
12analog	**O**	S	D	G	T	F	T	S	E	L	S	R	L	R	E	G	A	R	L	Q	R	L	L	Q	G	L	V
13analog	H	S	D	**P**	T	F	T	S	E	L	S	R	L	R	E	G	A	R	L	Q	R	L	L	Q	G	L	V
14analog	H	S	D	**Y**	T	F	T	S	E	L	S	R	L	R	E	G	A	R	L	Q	R	L	L	Q	G	L	V
15analog	H	S	D	G	T	F	T	S	E	L	S	R	L	**Q**	**D**	**S**	A	R	L	Q	R	L	L	Q	G	L	V
16analog	H	**V**	D	G	**R**	F	T	S	E	**F**	S	R	**A**	R	E	**GSA**	**A**	**I**	**R**	**K**	**I**	**I**	**N**	**S**	**A**	**L**	**A**
17analog	H	S	D	G	**L**	F	T	S	E	**Y**	S	**K**	**M**	R	E	**GNA**	**Q**	**V**	**Q**	**V**	**K**	**F**	**I**	**Q**	**N**	**L**	**M**
18analog	H	S	D	G	T	F	T	S	E	L	S	R	L	R	E	**GS**	**A**	**V**	**A**	**R**	**S**	**F**	**T**	**N**	**A**	**V**	**L**
19analog	H	S	D	G	T	F	T	S	E	L	S	R	L														
20analog														R	E	G	A	R	L	Q	R	L	L	Q	G	L	V

Peptide sequence alignment of all the peptide analogs of hSCT with modification highlighted in yellow.

GPCRs possess a similar topology and activation mechanism [48, 49]. Receptor-ligand interactions give rise to structural changes that result in multiple conformations, which is evident from the active and the inactive states [60, 61]. Recent studies have shown both interesting and complex processes of GPCR ligand responses, with different signaling outcomes upon activation [62]. The pharmacophores of hSCT are spread throughout the native ligand, as a result, the binding between the receptor and the SCT analogs could not be established due to instability/inefficiency. For class B GPCRs, it was hypothesized that the Ct region of the ligand is involved in initial receptor binding, and the Nt region is involved in receptor activation [23]. In summary, both *in vitro* and *in silico* studies indicate that small modifications in either the Ct or Nt of SCT result in complete abolishment of their activity in activating the receptor. Analog 15 (rSCT) has modification in the center of the peptide, and hence remains active. In analyzing the binding energy score, the main deviation was in the repulsive van der waals forces, and these forces were low in hSCT, rSCT, hVIP, hPACAP while all other peptide analogs have very high values (Table 6). Van der waals force is the sum of the attractive and repulsive force between molecules caused by fluctuating polarization of nearby particles [63]. The net total of van der waals forces can be attractive or repulsive [64]. The repulsive van der waals force in the peptide analogs (excluding rat secretin) were significantly elevated which is the main reason for the decrease in their binding affinities. Analyses of the docked structures by ligPlot+v.1.4.5 [41] reveal changes in the ligand-receptor interactions for all the analogs **(Figure E in** S1 File**)** due to changes in the secondary structures arising from amino acid substitutions. It may be augmented that due to the small size of the ligand, minor modification can result in dramatic changes to its structure, resulting in loss of function. It was believed that the C-terminal of secretin is crucial to its initial interaction with the receptor while the N-terminal for subsequent activation of the receptor. Nt (activation region) modified analogs with the intact Ct should theoretically be able to interact with the receptor and may function as antagonists, but none of these Nt-modified analogs could bind or affect the cAMP response of hSR. In our study, modifications of either the N- or the C-terminal resulted in a loss of function as well as loss of affinity for the receptor, shown though cAMP and FRET studies as well as in virtual docking. Consistently, we have shown that both termini of the peptide play important roles in binding with hSR.

**Table 6:** Expanded solution table of the docking files.

Peptide	glob	aVdW	rVdW	ACE	inside	aElec	rElec	laElec	lrElec	HB	piS	catpiS	aliph
hSCT	-11.53	-26.35	9.03	7.94	11.53	0.00	0.00	0.00	0.00	0.00	0.00	0.00	0.00
hVIP	-9.51	-14.44	5.23	-1.80	15.50	0.00	0.00	0.00	0.00	0.00	0.00	0.00	0.00
hPACAP	-6.31	-13.73	5.41	2.61	8.99	0.00	0.00	0.00	0.00	0.00	0.00	0.00	0.00
hGIP	10.63	-7.29	9.27	3.02	13.31	0.00	0.00	0.00	0.00	0.00	0.00	0.00	0.00
Analoge 1	7465.64	-48.23	9461.27	-23.70	9.85	0.00	0.00	0.00	0.00	0.00	0.00	0.00	0.00
Analoge 6	12.30	-5.59	1.32	8.49	8.65	0.00	0.00	0.00	0.00	0.00	0.00	0.00	0.00
Analoge 8	4.69	-12.99	7.65	2.73	19.55	0.00	0.00	0.00	0.00	0.00	0.00	0.00	0.00
Analoge 13	1010.29	-49.82	1367.15	-7.93	6.28	0.00	0.00	0.00	0.00	0.00	0.00	0.00	0.00
Analoge 15	-10.21	-15.70	4.90	-0.79	15.27	0.00	0.00	0.00	0.00	0.00	-0.50	0.00	0.00
Analoge 16	-1.38	-14.11	4.14	6.24	9.27	0.00	0.00	0.00	0.00	0.00	0.00	0.00	0.00
Analoge 17	14.45	-9.12	4.91	7.43	17.59	0.00	0.00	0.00	0.00	0.00	0.00	0.00	0.00

Solution table of the hSR model docked files with respective ligands at the binding site

glob—Global Energy, the binding energy of the solution

aVdW, rVdW—softened attractive and repulsive van der Waals energy

ACE—atomic contact energy (ACE)

inside—insideness measure

aElec,rElec—attractive and repulsive short-range Coulomb electrostatics

laElec, lrElec—attractive and repulsive long-range Coulomb electrostatics

HB—hydrogen and disulfide bonds

piS—PI-PI stacking

catpiS—cation-PI stacking

aliph—aliphatic interactions

## Supporting Information

S1 File **Fig A of S1. Primary sequence alignment of HSR**. Depicts the primary sequence alignment of HSR fasta sequence with Class B N-terminal templates PACAP N-terminal (PDB 2JOD), VIPR N-terminal (PDB 2X57) and GLPR-1 N-terminal (PDB 3C5T). The NT constraints were preserved and aligned for HSR NT modeling. **Fig B of S1. The primary sequence is alignment hSR**. The primary sequence is aligned and highlighted in seven different colors depicting the TM1 to TM7. **Fig C of S1. Template alignment for the fused model of hSR.** Template alignment for the fused model of hSR. The highlighted regions in pink color, is the NT model to replace the region highlighted in blue color, are the NT overhang region of the TM model. The fused model sequence would be as shown in the consensus. **Fig D of S1. Agonistic and antagonistic response of the analogs.** 1A-21A shows the agonistic response of the analogs at various doses and 1B -21B shows the antagonistic response of the same analogs in the presence of 5 nM secretin. **Fig E of S1. Docking model with different peptides.** The binding between the receptor model and different ligands including hSCT and hGIP as positive and negative control respectively. Analog 1, 8 and 13 is used as a representation of single amino acid substitution. Analog 6 19 and 20 as representation as middle, N terminal and C terminal subunits. Analog 15 (rat secretin) is shown as representation of amino acid substitution at center which also was capable of activating the receptor.
(DOCX)
