# Correction: Structure-Activity Relationship Studies of N- and C-Terminally Modified Secretin Analogs for the Human Secretin Receptor

**DOI:** 10.1371/journal.pone.0165770

**Published:** 2016-10-26

**Authors:** Kailash Singh, Vijayalakshmi Senthil, Aloysius Wilfred Raj Arokiaraj, Jérôme Leprince, Benjamin Lefranc, David Vaudry, Ahmed A. Allam, Jamaan Ajarem, Billy K. C. Chow

There are errors in the Funding section. The correct funding information is as follows: The present study was supported by the Hong Kong Government Research Grant Council grant, Collaborative Research Fund CRFHKU6/CRF/11G and Hong Kong University 764812M to B.K.C.C. & International Scientific Partnership Program ISPP at King Saud University through ISPP# 008.
