# Supplementary material for: Structure-Activity Relationship Studies of N- and C-Terminally Modified Secretin Analogs for the Human Secretin Receptor
Source: PLoS One. 2016 Mar 1;11(3):e0149359. doi: 10.1371/journal.pone.0149359 (PMC4773067; doi:10.1371/journal.pone.0149359)
Supplement: S1 File — Fig A of S1. Primary sequence alignment of HSR. Depicts the primary sequence alignment of HSR fasta sequence with Class B N-terminal templates PACAP N-terminal (PDB 2JOD), VIPR N-terminal (PDB 2X57) and GLPR-1 N-terminal (PDB 3C5T). The NT constraints were preserved and aligned for HSR NT modeling. Fig B of S1. The primary sequence is alignment hSR. The primary sequence is aligned and highlighted in seven different colors depicting the TM1 to TM7. Fig C of S1. Template alignment for the fused model of hSR. Template alignment for the fused model of hSR. The highlighted regions in pink color, is the NT model to replace the region highlighted in blue color, are the NT overhang region of the TM model. The fused model sequence would be as shown in the consensus. Fig D of S1. Agonistic and antagonistic response of the analogs. 1A-21A shows the agonistic response of the analogs at various doses and 1B -21B shows the antagonistic response of the same analogs in the presence of 5 nM secretin. Fig E of S1. Docking model with different peptides. The binding between the receptor model and different ligands including hSCT and hGIP as positive and negative control respectively. Analog 1, 8 and 13 is used as a representation of single amino acid substitution. Analog 6 19 and 20 as representation as middle, N terminal and C terminal subunits. Analog 15 (rat secretin) is shown as representation of amino acid substitution at center which also was capable of activating the receptor. (DOCX) [file pone.0149359.s001.docx]

**Supplementary fig. A:**

**
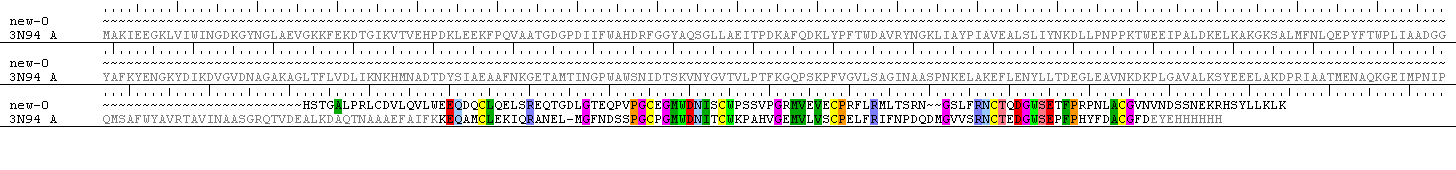
**

**Supplementary fig. A:** Depicts the primary sequence alignment of HSR fasta sequence with Class B N-terminal template. The NT constraints were preserved and aligned for HSR NT modeling.

**Supplementary fig. B:**

**
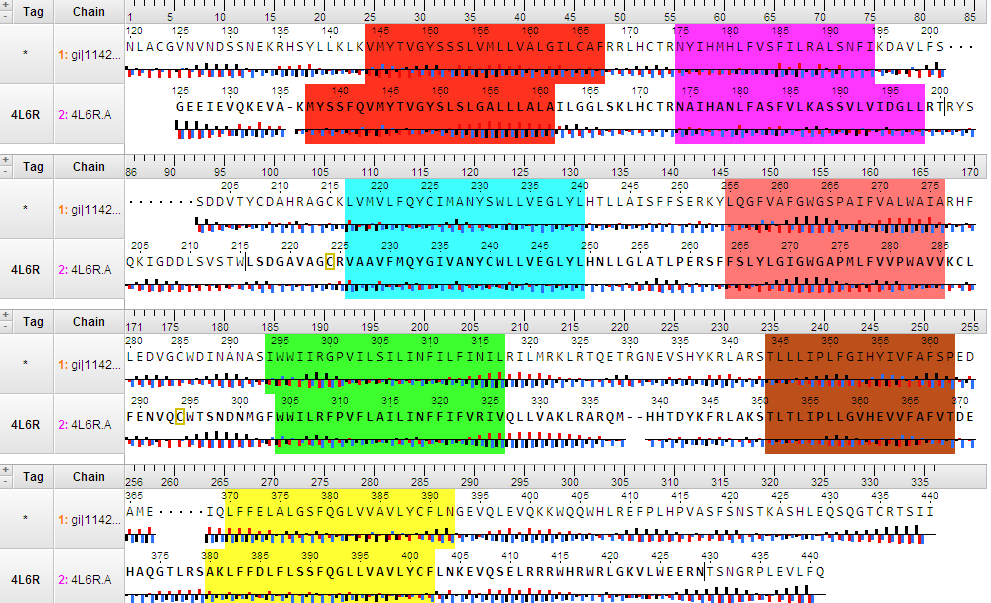
**

**Supplementary fig. B:** The primary sequence is aligned and highlighted in seven different colors depicting the TM1 to TM7.

**Supplementary fig. C:**

**
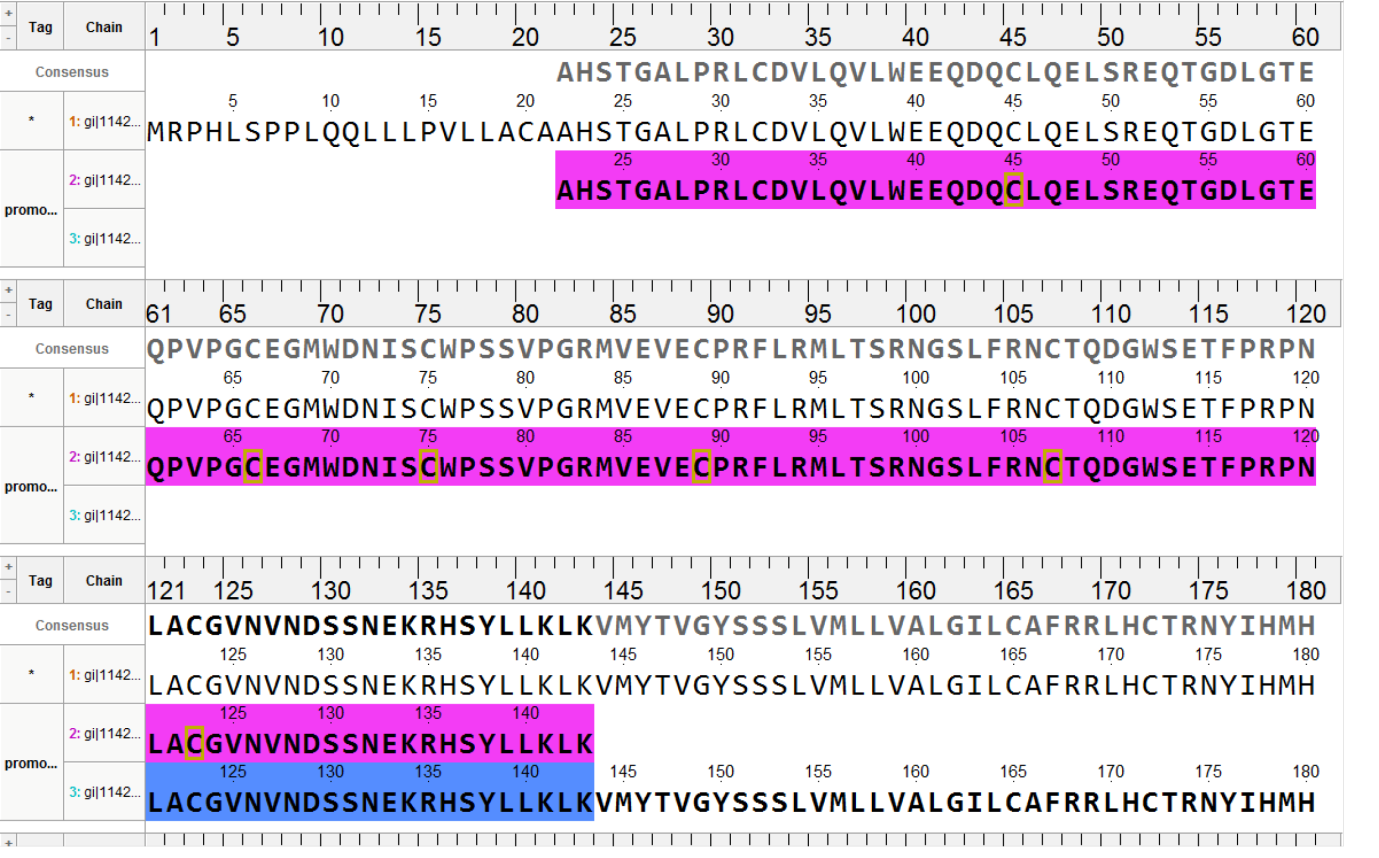
**

**Supplementary fig. C:** Template alignment for the fused model of HSR. The highlighted regions in pink color, is the NT model to replace the region highlighted in blue color are the NT overhang region of the TM model. The fused model sequence would be as shown in the consensus.

**Supplementary fig. D:**

**Analogs Analogs + 5 nM secretin**

| 1A   | 1B   |
| --- | --- |
| 2A   | 2B   |
| 3A   | 3B   |
| 4A   | 4B   |
| 5A   | 5B   |
| 6A   | 6B   |
| 7A   | 7b   |
| 8A   | 8B   |
| 9A   | 9B   |
| 10A   | 10B   |
| 11A   | 11B   |
| 12A | 12B   |
| 13A   | 13B |
| 14A | 14B |
| 15A   | NIL |
| 16A   | 16B |
| 17A   | 17B |
| 18A | 18B   |
| 19A   | 19B   |
| 20A   | 20B   |
| 21A   | 21B   |

**Supplementary fig.D:** 1A-21A shows the agonistic response of the analogs at various doses and 1B -21B shows the antagonistic response of the same analogs in the presence of 5 nM secretin.

**Supplementary fig. E**

**: a (**Model and hSCT)


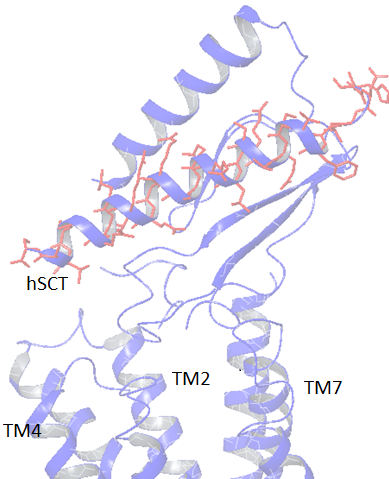


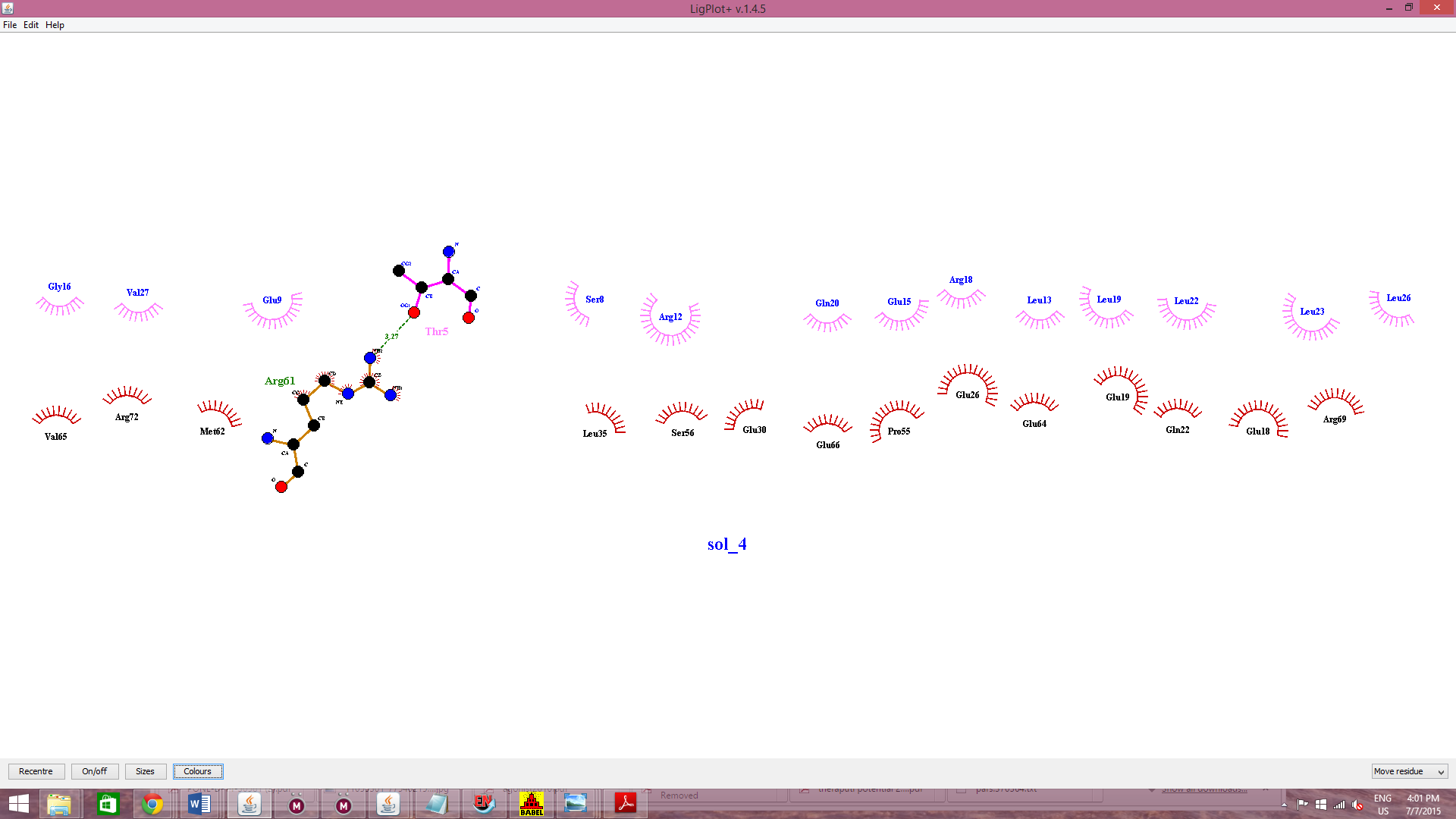


b (GIP and mode)l


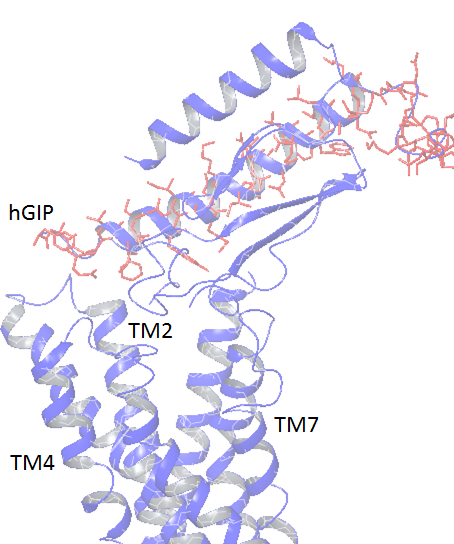


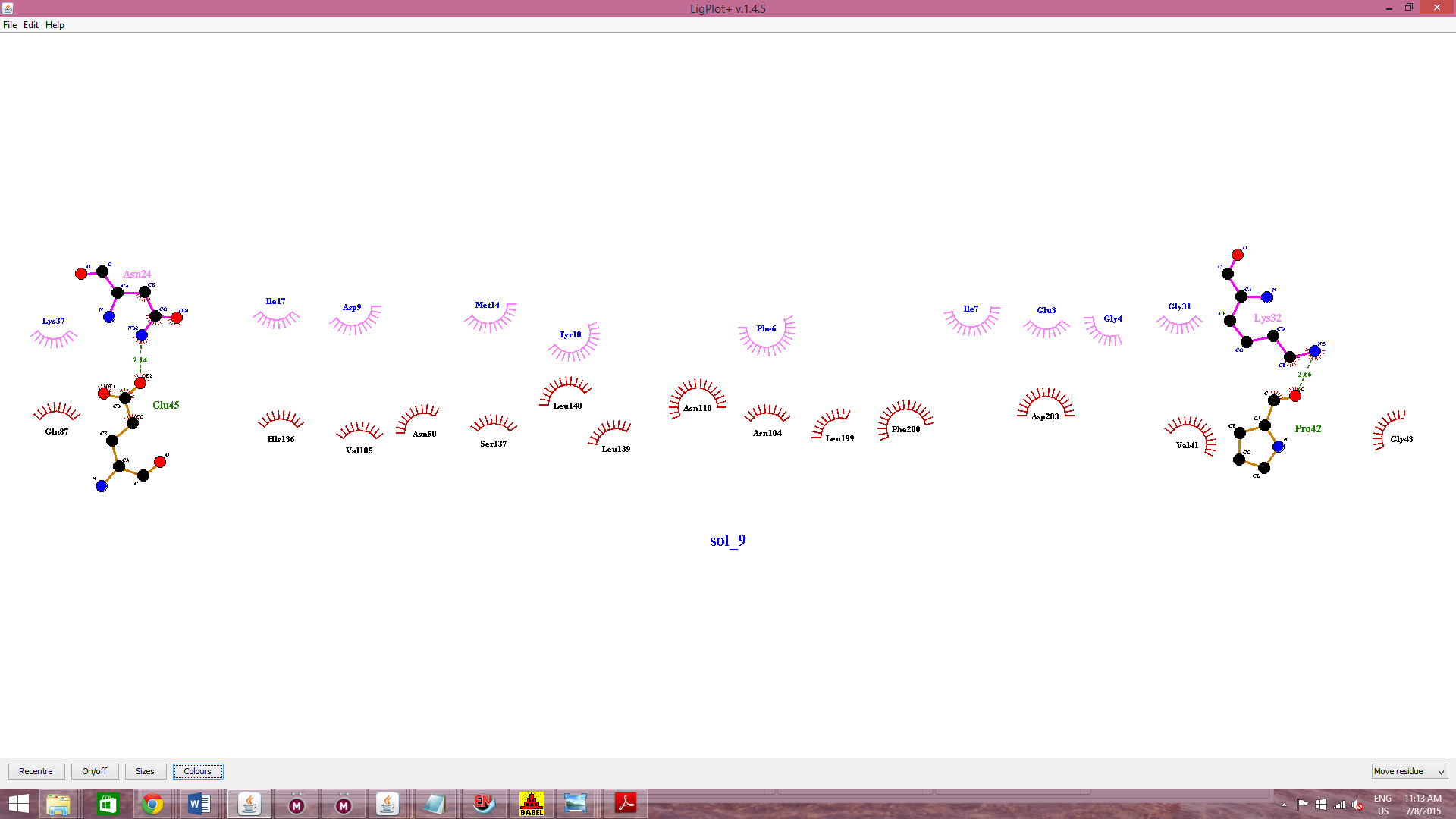


c (Analge1 and model)


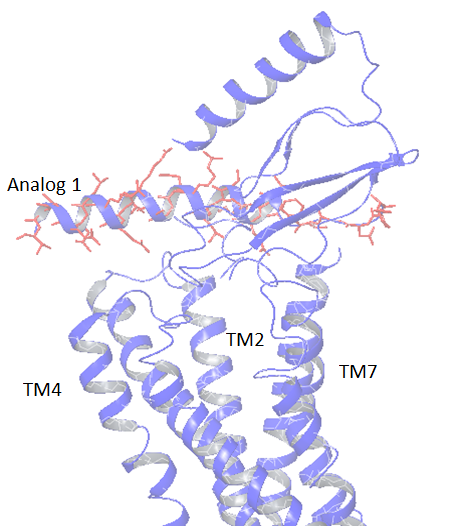


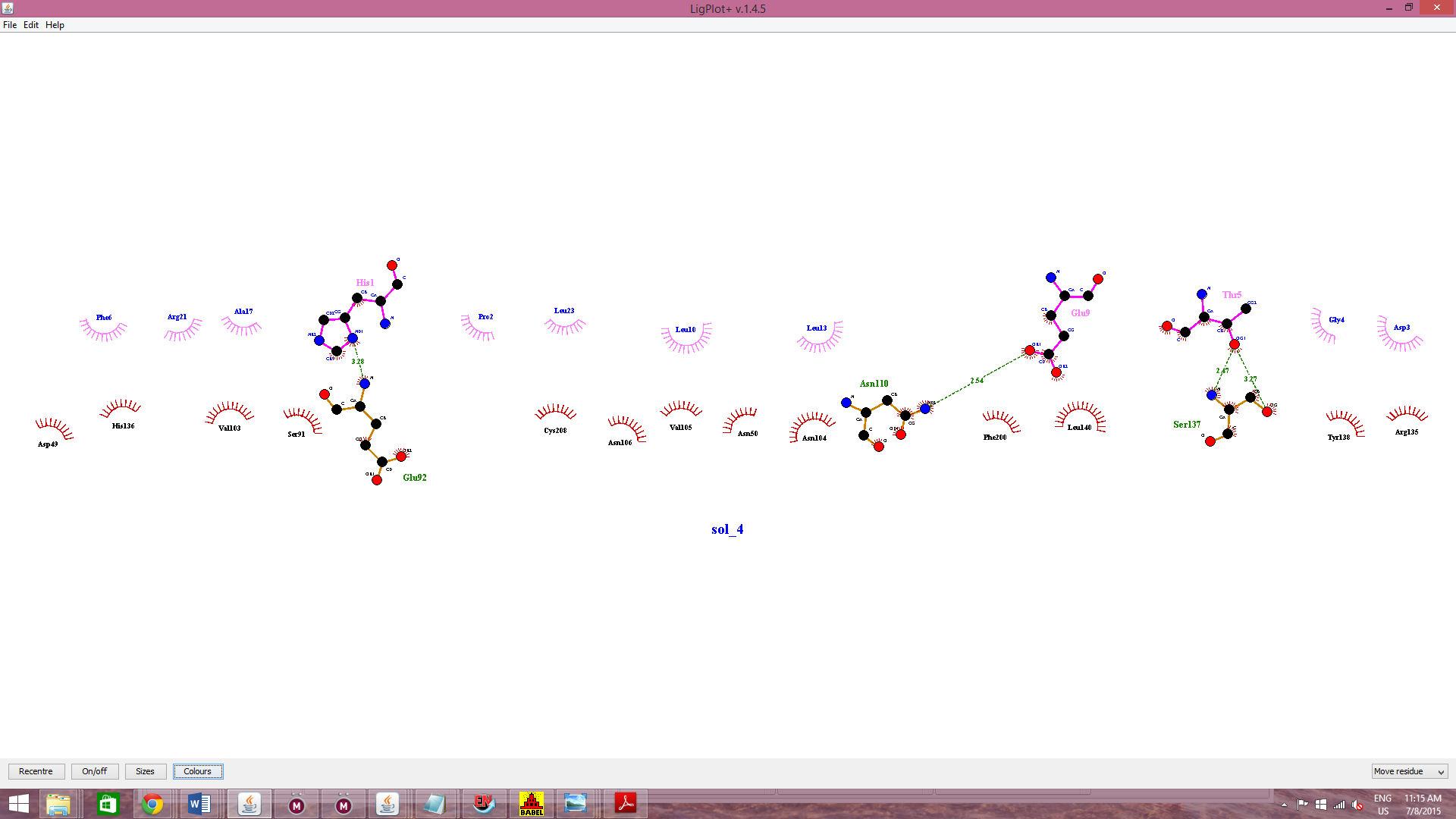


d (Mode and analog 6)


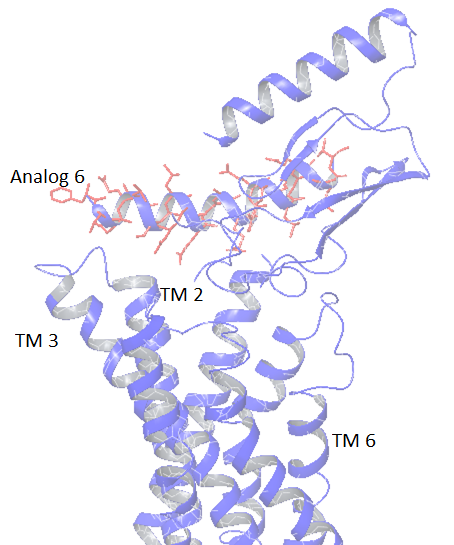


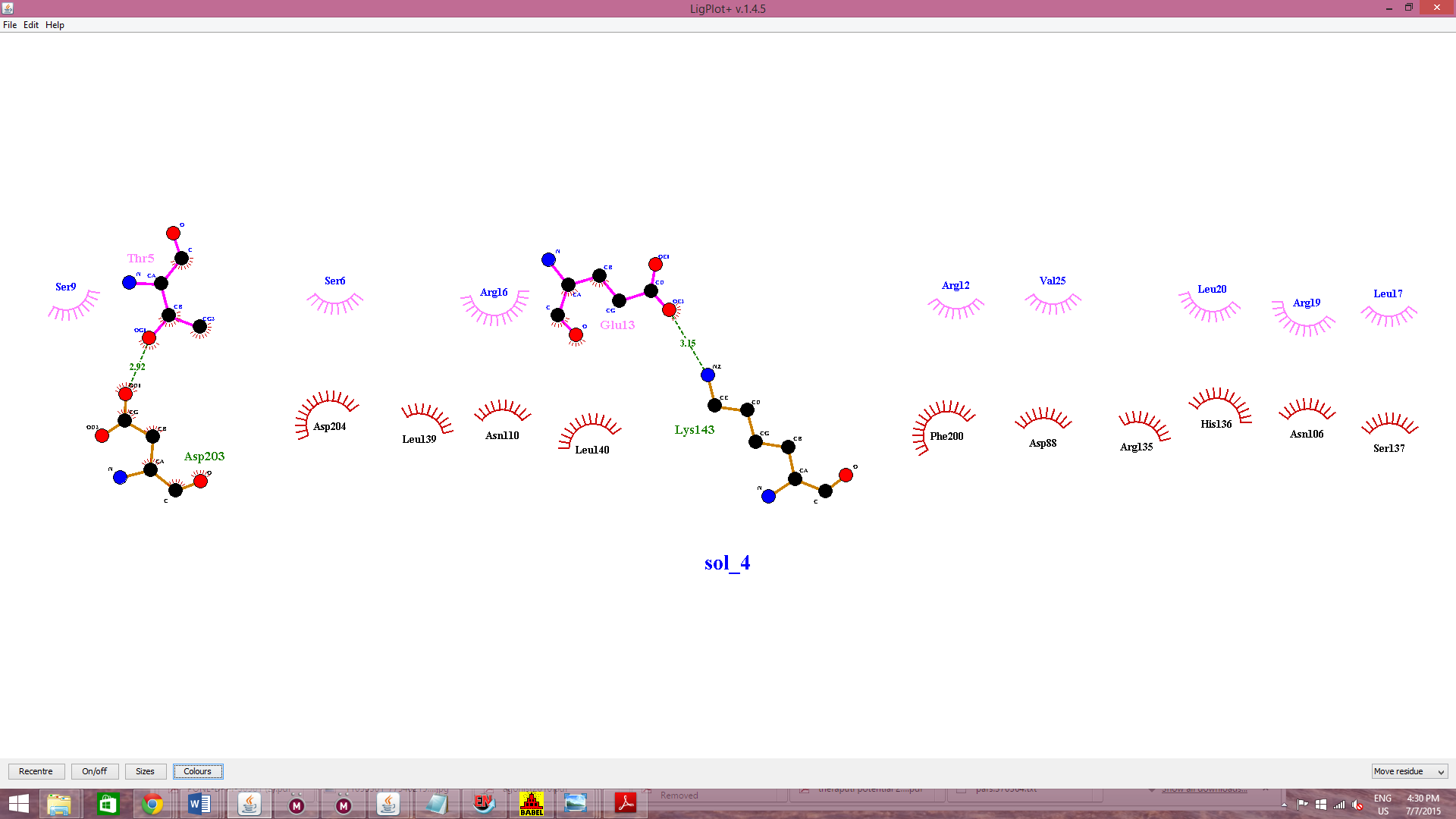


e (Model and analog 8)


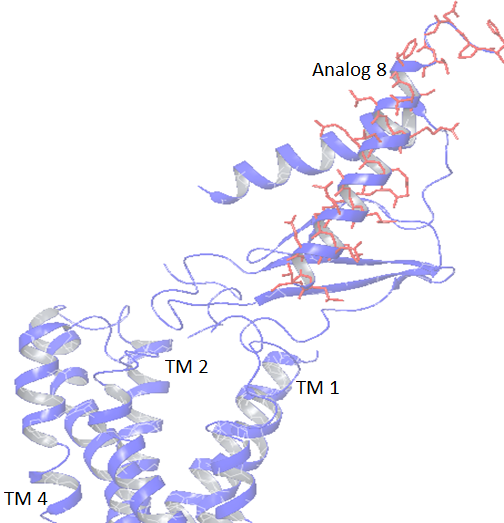


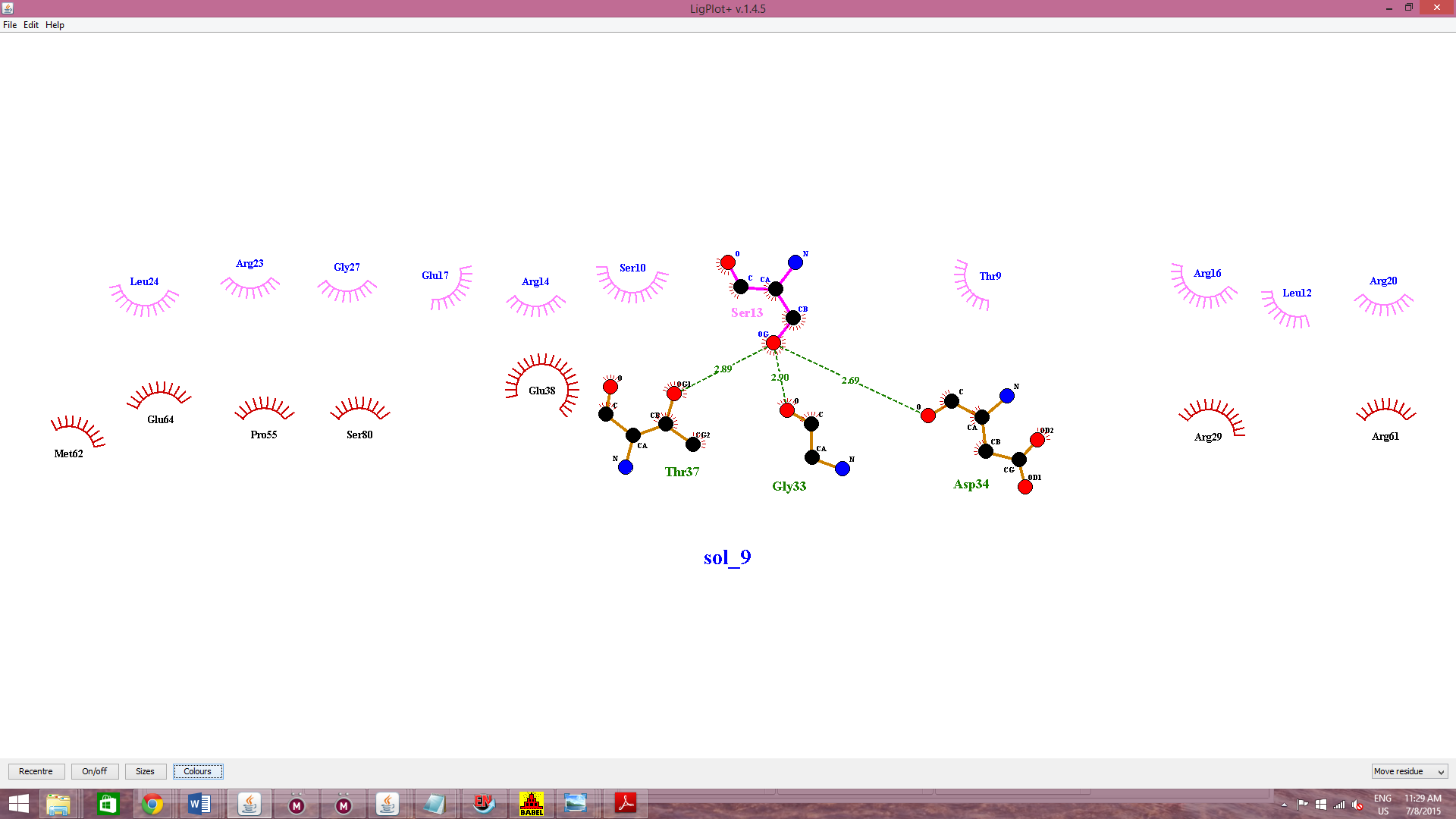


f (Analoge13 and model)


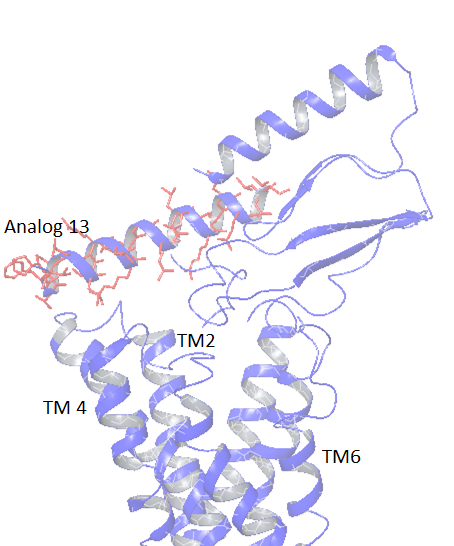


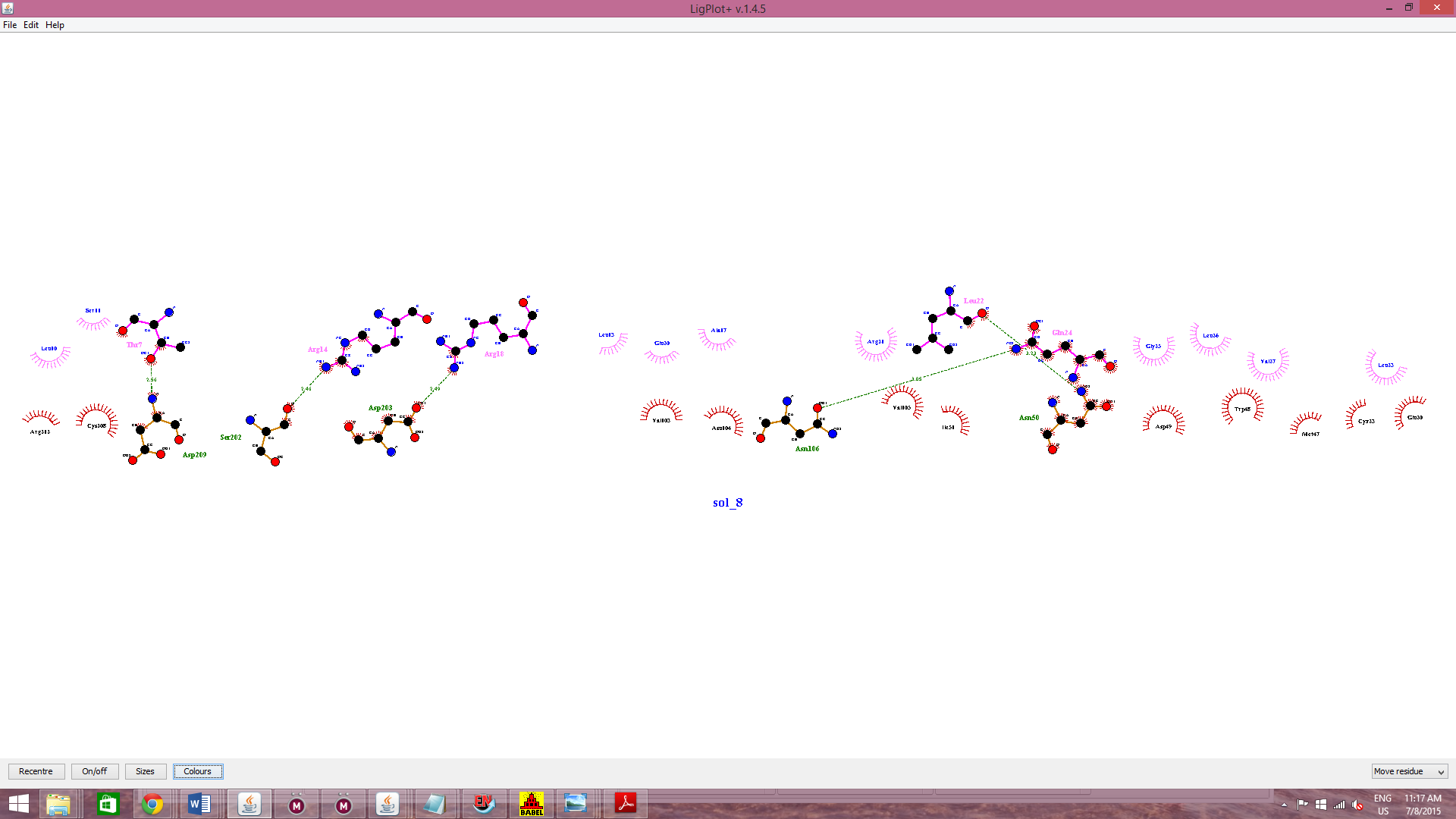


g (Analog 15 and model)


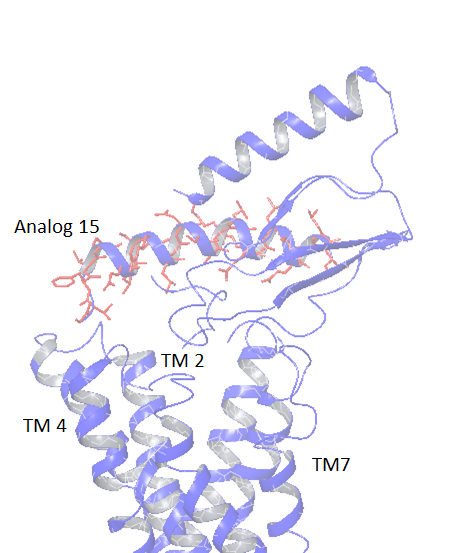


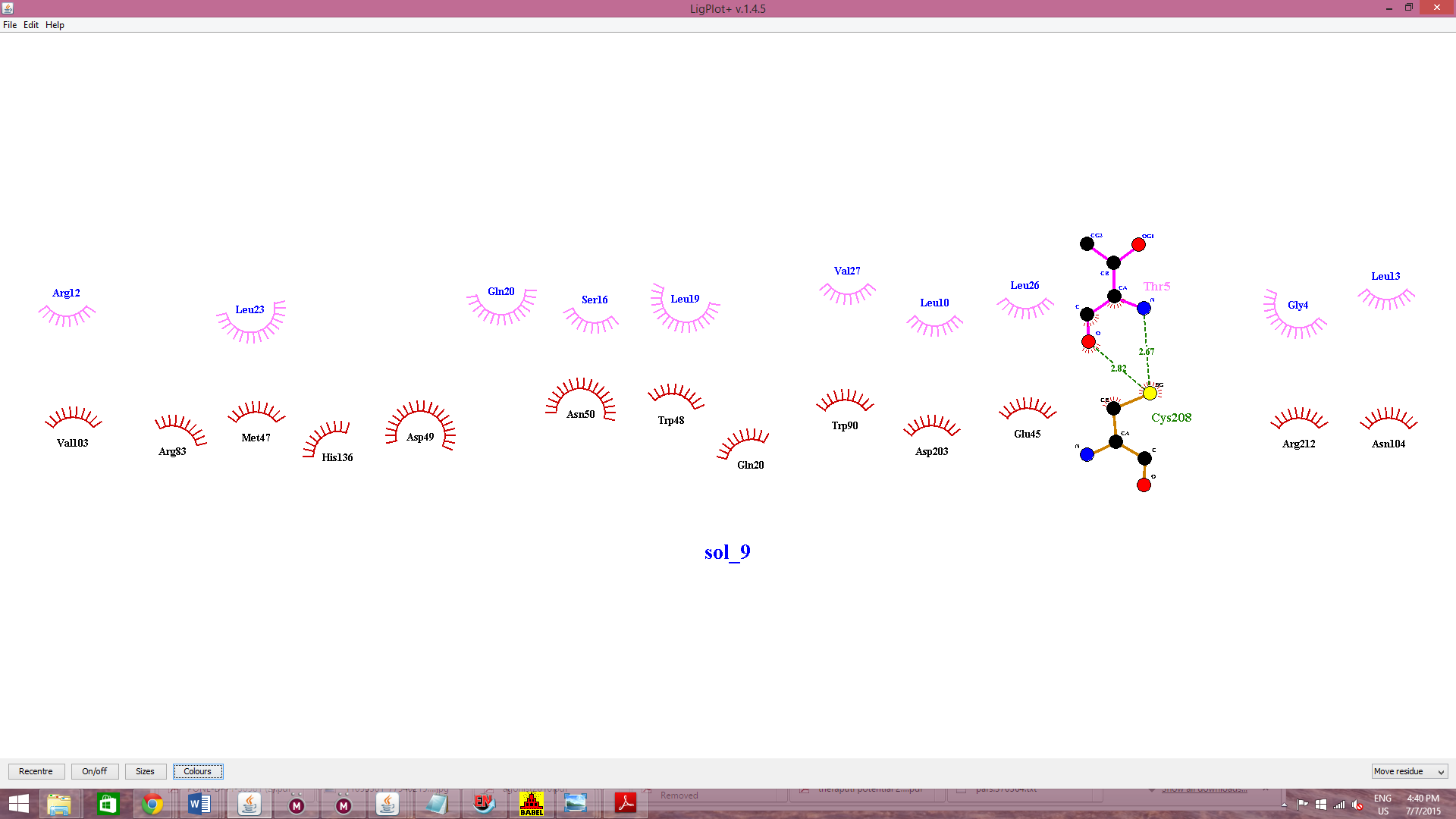


**Supplementary fig. E:** The binding between the receptor model and different ligands including hSCT and hGIP as positive and negative control respectively. Analog 1, 8 and 13 is used as a representation of single amino acid substitution. Analog 6 19 and 20 as representation as middle, N terminal and C terminal subunits. Analog 15 (rat secretin) is shown as representation of amino acid substitution at center which also was capable of activating the receptor.
